# Reduced number and impaired function of circulating progenitor cells in patients with systemic lupus erythematosus

**DOI:** 10.1186/ar2283

**Published:** 2007-08-31

**Authors:** Jan Renier AJ Moonen, Karina de Leeuw, Xavier J Gallego Y van Seijen, Cees GM Kallenberg, Marja JA van Luyn, Marc Bijl, Martin C Harmsen

**Affiliations:** 1 Department of Pathology and Laboratory Medicine, University Medical Center Groningen, University of Groningen, The Netherlands; 2 Department of Clinical Immunology, University Medical Center Groningen, University of Groningen, The Netherlands

## Abstract

Systemic lupus erythematosus (SLE) is associated with premature and accelerated atherosclerosis. Circulating progenitor cells (CPCs) are circulating bone-marrow derived cells that play an important role in the repair of vascular damage that underlies the development of atherosclerosis. The objective of this study was to determine the number and functionality of CPCs in patients with SLE. The study included 44 female SLE patients in an inactive stage of disease and 35 age-matched female controls. CPC numbers in the circulation were determined by FACS with monoclonals against CD14, CD34 and CD133. Peripheral blood-derived mononuclear cell (PBMNC) fractions were cultured in angiogenic medium. The endothelial-like phenotype was confirmed and the colony forming unit (CFU) capacity, migratory capacity and the potential to form clusters on Matrigel were determined. Expression of apoptosis inhibiting caspase 8L was analyzed in PBMNCs and CPCs by gene transcript and protein expression assays. The number of CD34–CD133 double-positive cells (*P *< 0.001) as well as the CFU capacity (*P *= 0.048) was reduced in SLE patients. Migratory activity on tumor necrosis factor-α tended to be reduced in patient CPCs (*P *= 0.08). Migration on vascular endothelial growth factor showed no significant differences, nor were differences observed in the potential to form clusters on Matrigel. The expression of caspase 8L was reduced at the transcriptional level (*P *= 0.049) and strongly increased at the protein level after culture (*P *= 0.003). We conclude that CPC numbers are reduced in SLE patients and functionality is partly impaired. We suggest these findings reflect increased susceptibility to apoptosis of CPCs from SLE patients.

## Introduction

Systemic lupus erythematosus (SLE) is a chronic systemic autoimmune disease. Premature and accelerated atherosclerosis, eventually leading to cardiovascular events, is a major cause of morbidity and mortality in patients with SLE [1-3]. Recently, it has been shown that patients with SLE have higher degrees of coronary artery calcification compared to age- and sex-matched controls with comparable classical risk factors [4,5]. Others have confirmed that traditional risk factors alone cannot fully account for the etiology of accelerated atherosclerosis in SLE patients [6-8]. These findings suggest a contributing role of the auto-immune disease itself in atherogenesis.

The response-to-injury model supports a key role for inflammation in the process of atherosclerosis [9]. As such, a functional and integral endothelial monolayer is critical to prevent the development of vascular disease. Damage that results in disintegration of the vascular endothelial monolayer is thought to be restored by either sprouting of preexisting endothelial cells or recruitment of circulating progenitor cells (CPCs) from the bone marrow [10,11]. Both monocyte-derived (CD14+) and hematopoietic stem cell-derived (CD34+ and CD133+) CPCs have been described [12,13].

In SLE patients, immune complex mediated inflammation increases stress on the vasculature, leading to an increased need of vascular repair [14]. The premature atherosclerosis present in SLE patients suggests that this repair is inadequate. This led us to hypothesize that the number and the functionality of CPCs are reduced in SLE patients.

## Materials and methods

### Characteristics of patients and controls

The study included 44 consecutive female SLE patients (aged 40 ± 12 years) who attended the out-patient clinic of the University Medical Center Groningen and 35 healthy age-matched female subjects (aged 41 ± 12 years). All patients fulfilled at least four of the SLE classification criteria from the American College of Rheumatology (ACR), and were in an inactive stage of disease defined as SLE disease activity index (SLEDAI) ≤4 [15,16]. At the time of inclusion, 18 of 44 patients had a SLEDAI score of 0, 17 had a score of 2 and 9 had a SLEDAI score of 4. Exclusion criteria were pregnancy, diabetes mellitus, cancer, presence of cardiovascular disease and use of HMG-CoA inhibitors. Cardiovascular disease was defined as a history of ischemic heart disease (ICD-9 classification 410–414), cerebrovascular accidents or peripheral vascular disease based on medical records. Median duration of disease was 96 months (interquartile (IQ) range 54 to 150 months). Patients had mild disease; the median damage index as measured by the SLICC/ACR was 0 (IQ range 0 to 1). Concerning medication, out of the 44 patients, 14 used antihypertensive drugs, 24 used prednisolone (median daily dose 5 mg, IQ range 5 to 10 mg), 13 used azathioprine (median daily dose 100 mg, IQ range 75 to 100 mg) and 23 used hydroxychloroquine (median daily dose 400 mg, IQ range 400 to 600 mg).

The local research ethics committee gave approval for the study and informed consent was obtained from each participant. The number of patients and controls included per assay are denoted below. Because of the low number of CPCs in the circulation of SLE patients and limitations in the amount of blood to be collected per patient, not all experiments could be conducted for each individual patient. However, patients and controls were matched to age in all experiments and there were no differences concerning the duration of disease, SLEDAI scores and medication between the subgroups used in the different assays.

### Isolation of peripheral blood-derived mononuclear cells

A 20 ml sample of heparinized venous blood was used for isolation of peripheral blood-derived mononuclear cells (PBMNCs). Samples were kept on ice and processed within 4 hours after collection. PBMNCs were isolated by density-gradient centrifugation (Lymphoprep, Axis-Shield).

### Fluorescence activated cell sorting

Freshly isolated PBMNCs (10 × 10^6^) from 20 patients and 20 controls were incubated with fluorescent-conjugated monoclonal antibodies (1:10) against CD14, CD34 (both from IQ Products, Groningen, The Netherlands) and CD133 (Miltenyi Biotec, Utrecht, The Netherlands). Cells were washed and resuspended in PBS. Measurements were performed on the FACSCalibur and CellQuest software was used for analysis (both from Becton and Dickinson, San Jose, CA, USA). The number of CD14+, CD34+ and CD133+ cells in the PBMNC fraction was recalculated to the number of cells per ml of peripheral blood.

### Overnight adhesion assay

PBMNCs were suspended in culture medium consisting of RPMI supplemented with 20% v/v fetal calf serum (both from BioWhittaker, Verviers, Belgium), 2 mM L-glutamine (GIBCO Products, Invitrogen, Breda, The Netherlands), 5 U/ml heparin (LEO Pharma, Ballerup, Denmark), 1% v/v PenStrep (Sigma, Zwijndrecht, The Netherlands), 50 μg/ml bovine brain extract (own isolate), 1 ng/ml vascular endothelial growth factor (VEGF)165 and 10 ng/ml basic fibroblast growth factor (both from Preprotech, Rocky Hill, NJ, USA). Cells (1 × 10^6^) were plated on human fibronectin/gelatin (1% v/v; Harbor Bio-products, Norwood, MA, USA) coated 24-well plates and incubated at 37°C, 5% CO_2 _overnight. Cells were gently washed with PBS and the number of attached cells was determined in ten high power fields per well. The average number of cells per high power field was calculated and multiplied to correct for the total well surface. The percentage was determined in relation to the initial 1 × 10^6 ^plated cells.

### Di-I-acetylated low density lipoprotein uptake and *Ulex *staining

PBMNCs were suspended in culture medium. Cells (4 × 10^5^) were plated on fibronectin-coated culture slides (Becton and Dickinson) and incubated at 37°C, 5% CO_2_; fresh medium was added on day 3. Cells were gently washed with PBS after seven days. Di-I-acetylated low density lipoprotein (Di-I-acLDL; Harbor Bio-products) was added to the slides (5 μg/ml) and incubated at 37°C, 5% CO_2 _overnight. Cells were washed with PBS and fixed using a 2% paraformaldehyde solution. Slides were washed with distilled water and incubated with 1 mg/ml *Ulex*-FITC (Sigma) in DAPI. Cells were mounted with Citifluor (Agar Scientific, Standsted, UK) and analyzed by fluorescence microscopy.

### Colony forming units assay

PBMNCs from ten patients and ten controls were suspended in culture medium. We plated 5 × 10^6 ^cells/well on 6 well plates coated as described above and incubated at 37°C, 5% CO_2_. Fresh culture medium was added on day 3. On day 8, the wells were gently washed with PBS and fresh culture medium was added. The numbers of colony forming units (CFU), characterized by a central cluster of cells surrounded by emerging cells, were counted manually as described by Hill and colleagues [17].

### Migration assay and Matrigel assay

On day 10 the culture medium was replenished. The plates were gently washed with PBS on day 14. Cells were dissociated with Accutase (PAA Laboratories GmbH, Cölbe, Germany). Cells from 10 patients and 10 controls were resuspended in DMEM (BioWhittaker) at 2 × 10^5 ^cells/ml for the migration assay and in culture medium at 5 × 10^5 ^cells/ml for the Matrigel assay.

The migratory capacity was measured using a chemotaxis chamber with 8 μm pore size filters (Neuro Probe, Gaitherburg, MD, USA). A concentration of 50 ng/ml proved optimal both for VEGF and tumor necrosis factor (TNF)-α. These concentrations were used in further experiments. Cultured CPCs (1 × 10^4^) were placed in the upper chamber. The lower chambers contained DMEM with recombinant VEGF, recombinant TNF-α (both from Preprotech) or DMEM only. After incubation at 37°C for 90 minutes, migrated cells were fixed and stained with Diff-Quik (Medion Diagnostics, Düdingen, Switzerland). Cells were counted manually in three high power fields per sample.

Per well of a 96 well plate, 45 μl of liquid Matrigel (Becton and Dickinson) was added and solidified at 37°C for 30 minutes. Cultured CPCs (1 × 10^5^) from 10 patients and 10 controls were added per matrigel-coated well and incubated at 37°C, 5% CO_2 _overnight. Cell clusters were counted in four high power fields per sample.

### Caspase 8(L) expression

The expression of caspase 8(L) was analyzed at the transcriptional and protein levels. To determine the different isoforms of caspase 8 mRNA, cDNAs from patient and control PBMNCs (*n *= 15 for both groups) were synthesized and amplified by RT-PCR: after 5 minutes of incubation at 94°C, RT-PCR was carried out for 60 s at 94°C, 45 s at 57°C, and 60 s at 72°C for 35 cycles using the following primers: caspase 8(L), sense 5'-aagcaaacctcggggatact-3' and anti-sense 5'-ggggcttgatctcaaaat ga-3'; and beta 2 microglobulin, sense 5'-gggttt catccatccgac-3' and anti-sense 5'-acggacggcatactcatc-3'.

For protein detection of caspase 8L using western blotting, 10 μg of protein sample was loaded on a SDS 12% polyacrylamide gel. Proteins were electrophoretically transferred onto nitrocellulose membranes (Protran, Schleicher and Schuell, Dassel, Germany). Nonspecific protein binding was blocked using PBS with 0.1% Tween 20 and 5% bovine serum albumin (Sigma). After incubation with anti-caspase 8 (Cell Signaling Technology, Danvers, MA, USA) 1:1,000 and anti-GAPDH (Abcam, Cambridge, UK) 1:2,000 overnight, the membranes were incubated with secondary antibodies 1:500 for 1 hour and alkaline-phospatase-conjugated tertiary antibodies 1:500 for 1 hour. BCIP/NBT alkaline phosphatase substrate (Bio-Rad, Veenendaal, The Netherlands) was used for detection.

The expression of caspase 8a and b and 8L was determined by densitometry (ImageJ software v1.37, NIH, USA) and the caspase 8L:caspase 8a+b ratio was calculated.

### Plasma levels of CRP and TNF-α

Plasma samples were obtained from 14 patients and 10 controls by centrifugation of heparinized venous blood at 800 G for 15 minutes.

C-reactive protein (CRP) levels were determined using commercially available antibodies (DakoCytomation, Glostrup, Denmark) in an ELISA as described before [18]. The detection limit of the assay was 0.1 mg/l. TNF-α levels were measured by ELISA, using a matched antibody pair and recombinant protein as standard from R&D Systems (Oxon, UK). After incubation and binding of biotinylated antibodies, the color reaction was performed with streptavidin-poly-HRP (Sanquin, Amsterdam, The Netherlands) and tetramethylbenzidin (Sigma). The detection level of TNF-α was 15 pg/ml.

### Statistical analysis

All data are presented as means ± standard error of the mean, unless stated otherwise. Statistical evaluations were performed with SPSS for Windows, version 12.0.2 (SPSS, Chicago, IL, USA). Data were evaluated with the Shapiro-Wilk test for normality; Student's *t*-test was used for normally distributed data, Mann-Whitney U for non-parametric data. Pearson's correlation coefficient was used to test for relations between complement levels and levels of antibodies against double-stranded (ds) DNA and the experimental outcomes (for example, number of CPCs and CFU formation). A probability value < 0.05 was considered statistically significant.

## Results

### Quantification of CPCs

**Figure 1:**
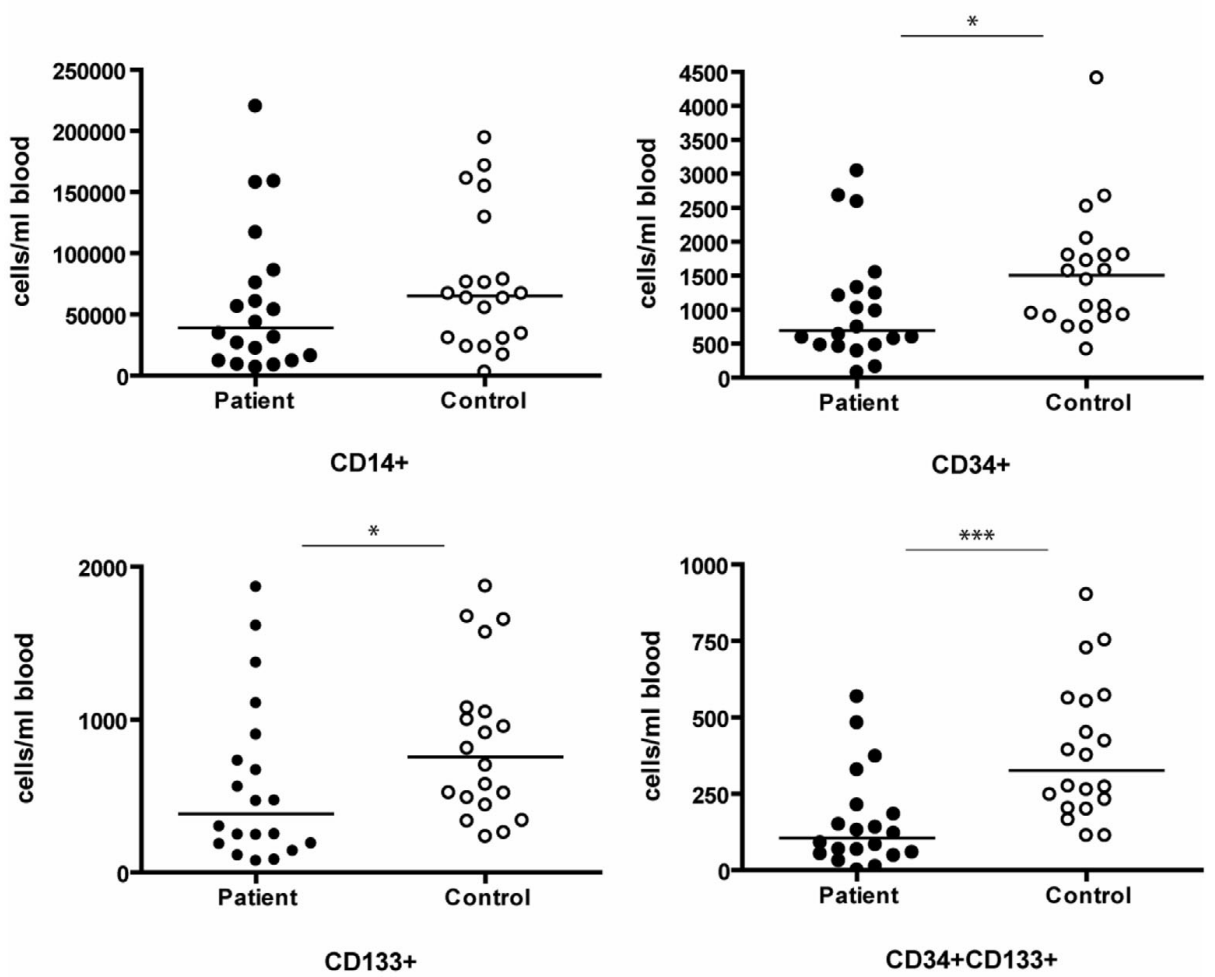
Number of CD14, CD34, and CD133 positive cells per ml of peripheral blood. The number of peripheral blood derived mononuclear cells from 20 patients and 20 controls that stained single-positive for CD14, CD34 and CD133 and co-stained for CD34 and CD133 were quantified by fluorescence activated cell sorting and are depicted here as the amount of positively stained cells per milliliter of peripheral blood (the line represents the median; *P < 0.05; **P < 0.01; ***P < 0.001).

The number of circulating CD34+/CD133+ CPCs was strongly decreased in patients (161 ± 35 versus 390 ± 50 cells/ml blood, *P *< 0.001). The number of PBMNCs that stained single-positive for CD34 and CD133 were also reduced (*P *= 0.008 and *P *= 0.015, respectively). Although the number of CD14+ monocytic cells was consistently lower in the patient group, the difference did not reach statistical significance (60,459 ± 13,384 versus 76,033 ± 12,634 cells/ml blood, *P *= 0.099; Figure 1)

### Overnight adhesion assay

**Figure 2:**
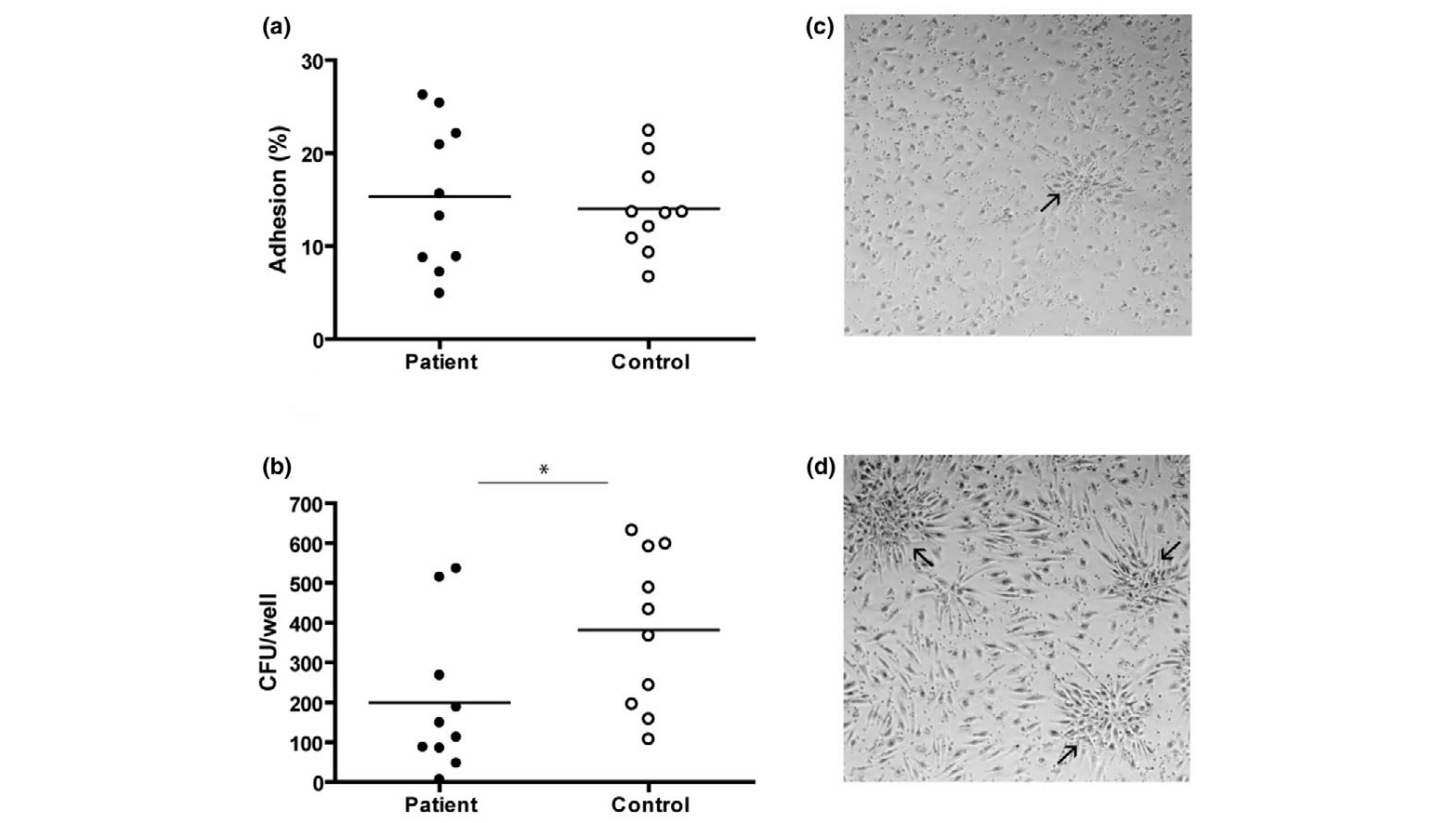
Adhesion assay and number and morphology of colony forming units (CFU). **(a) **Peripheral blood derived mononuclear cells (PBMNCs) from ten patients and ten controls were cultured overnight, percentages of attached cells were determined. **(b) **PBMNCs from ten patients and ten controls were cultured for one week after which CFU were formed and counted. In (a,b), the line represents the mean; *P < 0.05). Representative images of **(c) **a patient and **(d) **a control culture (the arrows point to a CFU). Notice the smaller size and the lower number of emerging cells when comparing patient CFU to control CFU.

The percentage of attached cells after overnight adhesion was determined and showed no differences between patients and controls (13.5 ± 2.1% and 13.8 ± 1.4%, respectively; Figure 2a).

### Confirmation of the endothelial phenotype of cultured mononuclear cells

PBMNCs were cultured in culture medium for seven days, after which the endothelial-like phenotype was confirmed by uptake of Di-I-acLDL and staining with *Ulex-*FITC. Nearly all cells stained double-positive, although occasionally, macrophage-like cells were seen. Patient and control PBMNCs showed similar staining (data not shown).

### Formation of CFU

Functionality of the cultured CPCs was determined in different ways, that is, CFU potential, migratory capacity and cluster formation capacity on Matrigel. After seven days of culture, central colonies of cells surrounded by emerging cells were formed. The number of these CFU was decreased in the patient population. (*P *= 0.048; Figure 2b). Although difficult to quantify, the CFU morphology differed between patient and control samples. CFU from patients were generally smaller in size and contained less emerging cells. Furthermore, the cultures of SLE patient PBMNCs contained less spindle shaped cells. Representative images are given below (Figure 2c,d).

### Migration of CPCs

**Figure 3:**
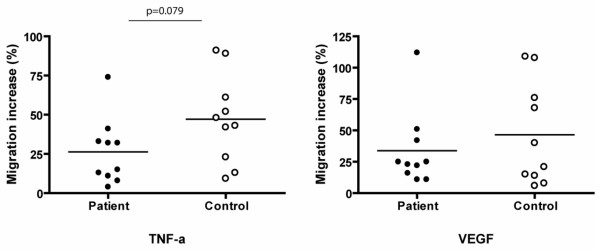
Migratory response of circulating progenitor cells (CPCs) to tumor necrosis factor (TNF)-α and vascular endothelial growth factor (VEGF). CPCs from ten patients and ten controls were cultured in angiogenic medium for 14 days, detached and placed in the upper chamber of a migration chamber. The lower chamber was filled with medium alone or with medium with either 50 ng/ml VEGF or 50 ng/ml TNF-α. Migration on VEGF and TNF-α was calculated as percentage of migration increase of CPCs compared to spontaneous migration on medium only. The line represents the mean.

After 14 days of culture in angiogenic medium, CPCs were detached and migration was induced on TNF-α and VEGF. The migratory activity was calculated as the percentage of migration increase on TNF-α or VEGF compared to the spontaneous migration on medium only. TNF-α driven migration tended to be reduced in patient CPCs (*P *= 0.079). Migration in response to VEGF showed no difference (*P *= 0.439; Figure 3).

### Cluster formation on Matrigel

**Figure 4:**
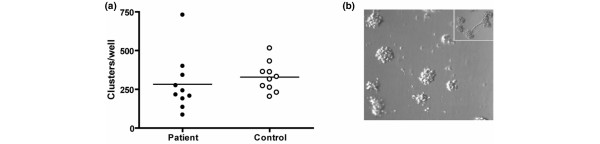
Cluster formation on matrigel. Circulating progenitor cells (CPCs) from ten patients and ten controls were cultured in angiogenic medium for 14 days. Cells were detached, resuspended in culture medium and 100,000 cells were added to matrigel coated wells. **(a) **After 18 hours the number of clusters containing more than ten cells were counted manually in three high power fields per sample. The line represents the mean. **(b) **A representative image of cluster formation on matrigel. The inset shows an example of a spouting CPC.

CPCs cultured for 14 days were plated on Matrigel-coated wells. Previously, cultured CPCs have been shown to spontaneously form sprouts during culture on Matrigel. Indeed, we also observed occasional sprouting in our cultures. As this was not always the case, we instead counted the clusters present in all of the cultures. The patient and control cultures showed similar counts (*P *= 0.482; Figure 4).

### Expression of caspase 8(L)

**Figure 5:**
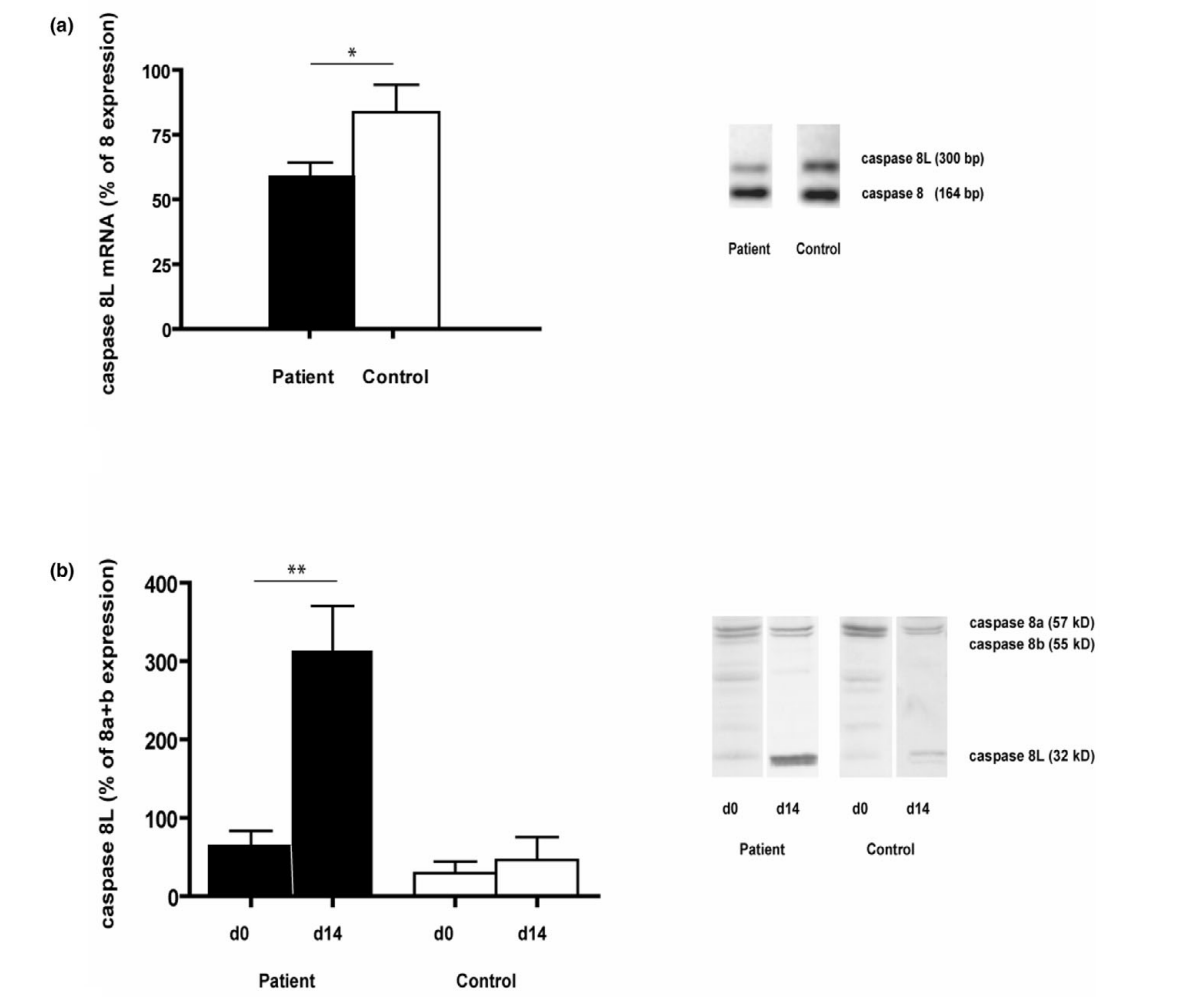
Caspase 8(L) expression. The expression of caspase 8(L) was analyzed at the transcriptional and protein expression levels (*n *= 15 for both groups and *n *= 5 for both groups, respectively). **(a) **At the transcriptional level, the uncultured peripheral blood derived mononuclear cells (PBMNCs) of systemic lupus erythemathosus patients showed a reduced caspase 8L:caspase 8 ratio. Representative images from patient and control bands are shown. **(b) **At the protein level, the ratios of the expression of caspase 8L:caspase 8a+b increased in the systemic lupus erythemathosus patient cells after culture for 14 days when compared to the uncultured PBMNC expression ratios, whereas the expression ratios remained the same in healthy control cells. Representative bands from the western blot are shown.

A reduced caspase 8L:caspase 8 mRNA ratio was found in freshly isolated PBMNCs of SLE patients (*P *= 0.049). On the protein level, the expression of caspase 8L compared to the combined expression of caspase 8a and 8b was similar in the PBMNC fractions of patients and controls (*P *= 0.343, *n *= 15 for both groups). To analyze changes in expression ratios after culture, we paired the ratios from freshly isolated PBMNCs and CPCs that were cultured for 14 days from 5 patients and 5 controls. The ratios in the control population remained the same, whereas the ratios in the cultured CPCs were strongly increased in the patient population (*P *= 0.6955 and *P *= 0.003, respectively; Figure 5).

### Plasma levels of CRP and TNF-α

Plasma from 14 patients and 10 controls was analyzed. No differences were found for CRP (5.329 ± 1.738 mg/l and 4.230 ± 1.993 mg/l, respectively, *P *= 0.684). TNF-α levels were below detectable levels in all controls and all but one patient (74 pg/ml).

### CPCs and clinical characteristics

To analyse the relation between serological disease parameters and experimental outcomes (number of CPCs, overnight adhesion capacity, CFU formation, migration on VEGF and TNF-α and cluster formation on matrigel) we determined the correlation between complement C3 and C4 levels, levels of antibodies to dsDNA and these experimental outcomes. No significant correlation was found between any of the serological disease parameters and outcomes (see Additional file 1). Next, we tested whether previous disease manifestations (as defined by the respective ACR criteria) were of influence on experimental outcomes. These outcomes in patients with, for example, renal involvement, serositis or arthritis were not different compared to outcomes in patients who never had these disease manifestations (data not shown). Finally, when patients were divided into subgroups depending on the use of immunosuppressive medication, that is, azathioprine or prednisolone, again no difference was found when experimental outcomes of patients using azathioprine or prednisolone were compared to those of patients not using these medications (see Additional file 2).

## Discussion

We found a reduced number of CPCs in SLE patients. Moreover, the CPCs were partly functionally impaired when compared to those from healthy controls. In concordance with several previous studies, we characterized a subpopulation of CPCs by double-positivity for CD34 and CD133 [19-21]. CD34 is expressed on hematopoietic stem cells of bone-marrow origin, but is also expressed on mature vascular endothelial cells. The second marker, CD133, is also expressed on hematopoietic progenitor cells, but not on mature endothelial cells [19,21]. During maturation of these cells, the expression of CD133 is lost. The combined expression of CD34 and CD133, therefore, defines a population of (immature) circulating progenitor cells. Because of the low number of CD34 and CD133 (double-)positive cells in the peripheral blood, we chose to enrich the cell populations by firstly isolating the total mononuclear cell fraction.

Another population of CPCs that acquires an endothelial phenotype *in vitro *is the monocyte [22,23]. CD14+ monocytes have also been shown to contribute to vascular repair *in vivo *[24-26]. Like the CD34–CD133 double-positive cell population, the number CD14+ cells was consistently reduced in the patient population.

The decrease in the number of circulating progenitor cells in patients with SLE is similarly seen in other diseases with vascular inflammatory components, for example, rheumatoid arthritis and chronic renal failure [27-29]. Recently, Westerweel and colleagues [30] also showed a decrease in the number of CD34+ cells in patients with SLE. Whilst the use of immunosuppressive medication can have a suppressive effect on the bone marrow, the use of low dose corticosteroids, such as in our patient group, has been shown to have no significant effect on CPC numbers [28]. The use of hydroxychloroquine might result in a mild increase in the number of CD34+ cells [30]. In our study population no significant differences were found for any of the outcome parameters when we compared patients using azathioprine or prednisolone to those who did not. The usage of HMG-CoA inhibitors was an exclusion criterion, as it has previously been reported to increase the mobilization of CPCs [31].

SLE is a condition with chronic or recurrent inflammation. Reduced mobilization and increased apoptosis, resulting in a shorter half-life of CPCs, can result from chronic exposure to inflammatory cytokines. We specifically focused on SLE patients with quiescent disease as this enabled us to study the chronic effects of the disease on CPC levels and functionality. Active disease is characterized by inflammatory activity. Whilst the effect on CPC numbers and functionality would be interesting to study in these patients, this is only temporal and, as such, will not reflect the chronic component, which is more likely to result in the long-term cardiovascular outcome in these patients. Increased levels of TNF-α have a suppressive effect on the bone marrow, resulting in reduced numbers of CPCs in the circulation [32]. Elevated levels of TNF-α have been described in the circulation of SLE patients [33,34]. In our study population we found no TNF-α elevation in all but one patient, thereby confirming the inactive stage of disease of these patients. However, in the bone marrow, where TNF-α is rarely present in healthy subjects, patients with SLE show a high expression of TNF-α mRNA [35]. TNF-α induces functional Fas on hematopoietic progenitor cells [36]. Studies conducted by Papadaki and colleagues [37] showed a reduced number of CD34+ cells in the bone marrow of SLE patients and a higher expression of Fas antigen in the CD34+ cell fraction compared to controls. This increase of CD34+/Fas+ double-positive cells correlated with a significantly higher number of apoptotic CD34+ cells [37]. Increased apoptosis of CD34+ cells in patients with SLE was confirmed by Westerweel and colleagues [30]. We hypothesized that the increased apoptosis might be caused by a decreased expression of caspase 8L. In 2000, Horiuchi and colleagues [38] reported a longer isoform of caspase 8, termed caspase 8L, which results from alternative splicing of the caspase 8 gene. While the splice form encodes the amino-terminal two repeats of the death effector domain (DED), it lacks the carboxy-terminal half of the proteolytic domain. Caspase 8L binds to the Fas-associating protein with death domain (FADD) and blocks the binding of caspase 8 to FADD by competiive inhibition [39]. Indeed, in hematopoietic stem cells and leukemic cells, the elevated expression of caspase 8L protects these cells from Fas-mediated apoptosis [40]. Our results show a reduced expression of caspase 8L in the PBMNC fractions at the mRNA level, which is in concordance with the findings from Horiuchi and colleagues [38]. At the protein level, no differences were observed in the PBMNC fractions of patients and controls. Interestingly, however, when we compared the expression ratio of caspase 8L:caspase 8a+b before culture with the expression ratio after culturing for 14 days, we observed an increase of caspase 8L expression in the cultured CPCs from SLE patients, whilst no difference was observed in the control CPCs. Apparently, during culture, CPCs were selected with more protection against Fas-mediated apoptosis. Whether this indicates the existence of subsets of CPCs that intrinsically differ with respect to apoptotic responsiveness or that CPCs can adapt to culture conditions remains to be investigated.

The proliferative capacity of CPCs from patients with SLE was reduced based on their decreased CFU potential. Morphologically, the patient CFU were smaller in size and contained less emerging, spindle-shaped cells. Van Beem and colleagues [41] have shown that these CFU and the spindle-shaped emerging cells are monocyte-derived CD14+ cells but are supported by CD14- cells. CD34+ hematopoietic stem cell-derived progenitor cells are suggested to play an important role in the regulation of CFU formation (US provisional patent P0025592). George and colleagues [42] found a correlation between reduced CFU counts and the reduction of adhesive properties of CPCs. One could logically consider the *in vitro *binding of CPCs to fibronectin as a reflection of the capacity of CPCs to bind to damaged endothelium *in vivo*. However, the overnight adhesion assay showed similar percentages of adhered cells for patients and controls, implying that the differences in CFU numbers are not due to reduced adhesive properties of the cells.

Migratory activity of cultured CPCs in response to TNF-α tended to be decreased in the patient group. Migration on VEGF showed no differences between patients and controls, nor were patient CPCs impaired in their capacity to form clusters on Matrigel. Clinical characteristics, that is, past disease manifestations, levels of complement factors or levels of antibodies against dsDNA did not show any relation to the experimental outcomes.

## Conclusion

Taken together, our data is largely in agreement with the recent study by Westerweel and colleagues [30]. However, we characterized CPCs by CD34 and CD133 double-positivity, confirmative for an immature progenitor cell. This prevents the false inclusion of CD34 and KDR double-positive mature endothelial cells which, as a result of vasculitis, might have detached from the vascular wall. Furthermore, as the authors state, the majority of PBMNCs that form endothelial-like cells *in vitro *are CD14+ and because CD14+ cells also contribute to vascular repair [25] we also analyzed the CD14+ CPC fraction. SLE patient cells were impaired in their capacity to form CFU and were less viable, resulting in lower numbers of cells after 14 days of culture (data not shown). The number of circulating CD14+ cells was reduced in SLE patients, although less pronounced than the number of circulating CD34–CD133 double positive cells. Along with the caspase 8L data, these data are suggestive for an apoptosis related problem that is not specifically limited to CD34+ cells but involves CD14+ cells as well. However, this clearly remains as a subject for further studies.

The CFU potential after seven days of culture was reduced in the patient population; after an additional seven days of culture, however, no significant differences were observed between the functionality of patient and control CPCs. We therefore propose that CPCs from SLE patients are not intrinsically impaired in their functionality, but are impaired due to extrinsic factors. Culturing of CPCs in defined conditions for 14 days has resulted in near to normal functionality of these cells. This can be beneficial for cell therapy purposes, in which CPCs are firstly cultured before they are re-administered to the patient. However, amelioration of the *in vivo *environment would be necessary to improve the CPC biology of SLE patients and open the way to prevent the accelerated atherosclerosis that characterizes many of these patients [6].

Additional file 1

Additional file 1 is a PDF containing a table showing the correlations of serological disease parameters with experimental outcomes. Using Pearson's correlation coefficient, no relations were found between levels of complement factors C3 and C4 or levels of antibodies against dsDNA and the experimental outcomes.

Additional file 2

Additional file 2 is a PDF containing a table showing the effect of immunosuppresive medication use on experimental outcomes. The use of immunosuppresive medication, that is, azathioprine and prednisolone, had no influence on the experimental outcomes. No differences were found between patients using azathioprine or prednisolone and those who did not.

## Abbreviations

ACR = American College of Rheumatology; CFU = colony forming unit; CPC = circulating progenitor cell; CRP = C-reactive protein; DI-I-acLDL = Di-I-acetylated low density lipoprotein; DMEM = Dulbecco's modified Eagle's medium; ds = double-stranded; ELISA = enzyme-linked immunosorbent assay; FITC = fluoro-isothiocyanate; IQ = interquartile; PBMNC = peripheral blood derived mononuclear cell; PBS = phosphate-buffered saline; SLE = systemic lupus erythemathosus; SLEDAI = SLE disease activity index; TNF = tumor necrosis factor; VEGF = vascular endothelial growth factor.

## Competing interests

The authors declare that they have no competing interests.

## Authors' contributions

JM was in charge of most of the experimental work, data analysis and drafting of the manuscript. KL was in charge of the recruitment of patients and their demography and performed the ELISA measurements. XS participated in the culturing PBMNCs and functionality assays. CK participated in the design of the study and helped to draft the manuscript. ML participated in the design and coordination of the study and helped to draft the manuscript. MB provided the clinical background, was in charge of the recruitment of patients and participated in design and drafting of the manuscript. MH participated in the design and coordination of the study and helped to draft the manuscript. All authors read and approved the final manuscript.
